# Case report: A rare case of retroperitoneal kaposiform hemangioendothelioma with spinal involvement without abnormal platelet count in ^18^F-FDG PET/CT

**DOI:** 10.3389/fmed.2022.946477

**Published:** 2022-08-11

**Authors:** Yongkang Qiu, Zhao Chen, Qi Yang, Wenpeng Huang, Lele Song, Yan Fan, Lei Kang

**Affiliations:** Department of Nuclear Medicine, Peking University First Hospital, Beijing, China

**Keywords:** kaposiform hemangioendothelioma, kasabach-merritt phenomenon, spinal involvement, sirolimus, PET/CT

## Abstract

Kaposiform hemangioendothelioma (KHE) is a rare vascular neoplasm that mostly appears in infancy or early childhood. Most KHE occurred on the limbs and trunk with cutaneous lesions. Approximately 12% of KHE patients manifested as deep masses and spinal involvement is extremely rare. KHE may develop into life-threatening thrombocytopenia and consumptive coagulopathy, known as the Kasabach-Merritt phenomenon (KMP), especially in patients with retroperitoneal involvement. The thrombocytopenia is usually severe, with a median platelet count of 21 × 10^9^/L at the initial presentation of KMP. Here, firstly we described a case of a 13-month-old girl with KHE who presented the movement limitation of the lower extremity caused by spinal involvement with a normal platelet count. ^18^F-fluorodeoxyglucose-positron emission tomography/CT (^18^F-FDG PET/CT) showed mildly elevated metabolism in the lesion, suggesting a probably low-grade malignant tumor. Then the patient was diagnosed with KHE by biopsy. After 6-month sirolimus monotherapy, the size of the retroperitoneal lesion was reduced significantly and the patient showed improvement in clinical symptoms. This case demonstrated the advantage of ^18^F-FDG PET/CT in the evaluation of disease activity in KHE and the possibility of using ^18^F-FDG PET/CT to guide therapy and prognostication.

## Introduction

Kaposiform hemangioendothelioma (KHE) is a rare vascular neoplasm that mostly appears in infancy or early childhood. The pathogenesis of KHE has not yet been discovered (1). The main pathological features of KHE are dysregulation of angiogenesis and lymphangiogenesis. The histologic hallmark of KHE is infiltrating, defined and confluent nodules of neoplastic spindled endothelial cells involving multiple planes of tissue, and the clusters of spindled tumor cells exhibit multiple slit-like vascular lumina, containing red blood cells (2). KHE can be classified into superficial, mixed, and deep lesions based on depth. Approximately 12% of KHE patients manifested as deep masses (3). The manifestations of KHE are variable, ranging from cutaneous lesions with variable performance to deep masses without cutaneous signs. KHE can be accompanied by a life-threatening complication Kasabach-Merritt phenomenon (KMP), which is characterized by thrombocytopenia, hypofibrinogenemia, and clotting factor consumption (3). Intrathoracic and retroperitoneal lesions are more likely to develop KMP because they are more expansive and infiltrative.

There is very little published research on ^18^F-fluorodeoxyglucose-positron emission tomography/CT (^18^F-FDG PET/CT) in KHE. Herein we report a case of retroperitoneal KHE with lower extremity mobility limitation caused by spinal involvement without abnormal platelet count. ^18^F-FDG PET/CT scan helped to find the retroperitoneal lesion with mildly elevated metabolism, which suggested low disease activity.

## Case description

A 13-month-old girl presented with lower extremity mobility limitation for 10 months. Left lower extremity weakness was first noted at 3 months of age, followed by pain when the left leg was straightened or extended. In the suspicion of bone disease, she underwent a non-contrast-enhanced CT scan that showed a pelvic mass with the destruction of the adjacent bone. Laboratory tests were normal and no obvious skin lesions were observed. The patient was diagnosed with immune thrombocytopenia at 7-month age, and the minimum platelet count was 38 × 10^9^/L. But her platelet counts gradually return to normal without special treatment. The patient performed bone puncture and flow cytometry to rule out hematological diseases.

**FIGURE 1:**
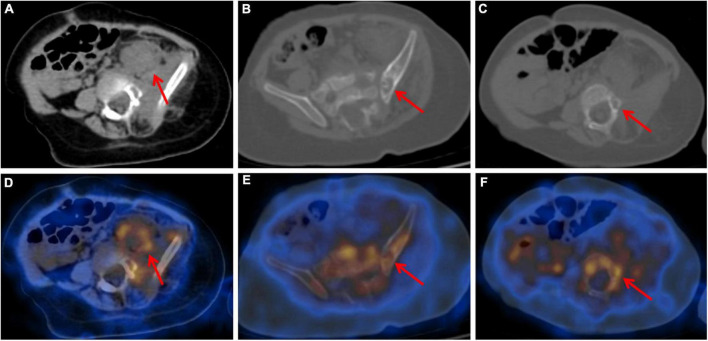
Axial CT **(A–C)** and fused PET/CT (**D–F**) images showed retroperitoneum mass with unevenly mildly increased metabolic uptake (**A,D**) and mixed lytic and sclerotic changes of left iliac bone (**B,E**) and L5 (**C,F**). Adjacent bones also presented mildly increased metabolic uptake.

**FIGURE 2:**
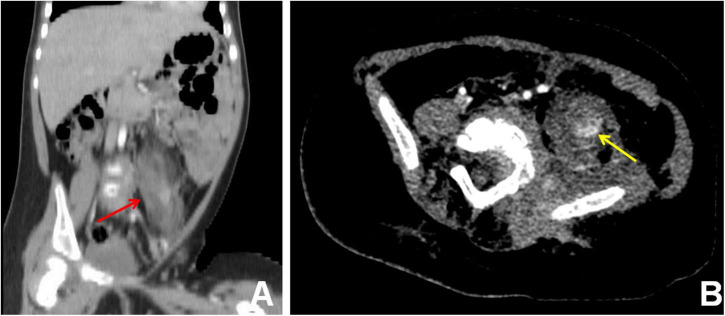
Contrast-enhanced CT scan, coronal view **(A)**, and axial view **(B)**, showed the ill-defined lesion of the retroperitoneum **(A)** (red arrow) and rich blood vessels **(B)** (yellow arrow) in the mass.

**FIGURE 3:**
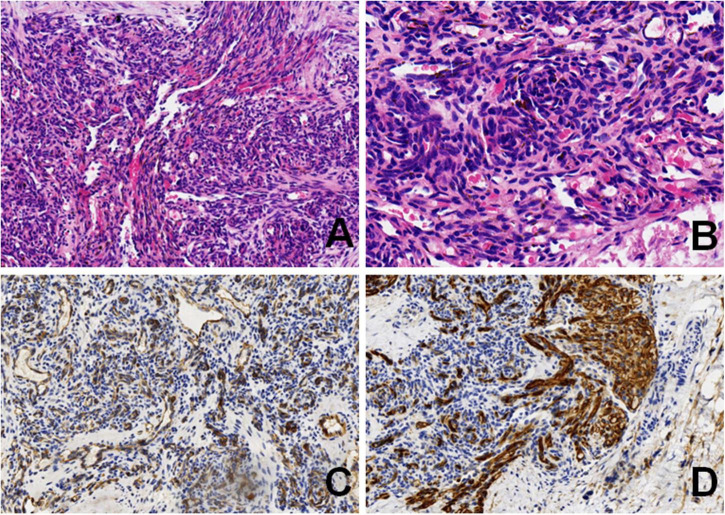
Photomicrograph showed irregular spindle tumor cell nodules in the lesion, containing a large number of red blood cells **(A)** (HE, original magnification × 10). Higher magnification showed a glomeruloid pattern of small central slit-like vessels **(B)** (HE, original magnification × 20). Neoplastic cells are positive for CD31 **(C)** (original magnification × 10), and D2-40 **(D)** (original magnification × 10).

To assess the patient’s whole-body condition and metabolically characterize the lesion, ^18^F-FDG PET/CT examination was further performed. It revealed unevenly mildly increased metabolic uptake within the already known mass in the left retro-peritoneum. The boundary of the lesion is not clear, with an approximate size of 3.3 cm × 2.5 cm and SUVmax of 1.7 (Figures 1A,D). L4, L5, and left iliac bone showed mixed lytic and sclerotic changes, SUVmax of 1.8 (Figures 1B,C,E,F). Left psoas, iliopsoas, erector spinae, and gluteus medius were enlarged when compared to contralateral side muscles, as also evident at a subsequent contrast-enhanced CT scan. Contrast-enhanced CT scan showed the ill-defined lesion (Figure 2A) and rich blood vessels in the mass (Figure 2B). There were no other abnormal CT features or ^18^F-FDG Uptake. Then the patient underwent a fine-needle biopsy of the retroperitoneal mass. Photomicrograph of the specimen showed there are many irregular hemangioma-like tumor cell nodules in the lesion, containing a large number of red blood cells (Figures 3A,B). The tumor cells were positive for Vimentin, CD31 (Figure 3C), CD34, ERG, SMA, and D2-40 (Figure 3D). Ki-67 staining showed the proportion of the positive tumor cells was about 1–2%. Upon pathologic examination with immunohistochemistry, KHE was diagnosed.

**FIGURE 4:**
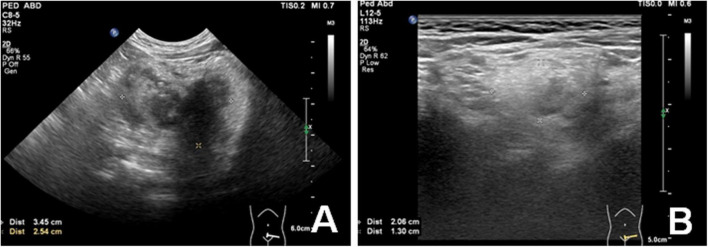
Ultrasound image showed a significant decreased (80%) volume of peritoneal mass before **(A)** and after 6-month Sirolimus monotherapy **(B)**.

Then, sirolimus.8 mg/day monotherapy was started. Blood drug concentration and blood routine were monitored regularly. After 3-month sirolimus monotherapy, abdominal ultrasound suggested a reduction of the lesion. Meanwhile, the clinical symptoms and signs were recovered soon. She can stand for about 20 s. Blood drug concentration showed a mildly low level (6.2 ng/ml), so the dose of sirolimus changed to 1 mg/day. After 6-month sirolimus monotherapy, the retroperitoneal mass was reduced by 80% (Figure 4) and the clinical symptoms almost disappeared. The patient is still receiving sirolimus monotherapy and regular follow-up. During sirolimus treatment, there were no obvious adverse reactions or abnormal laboratory tests. The patient’s platelet count has remained at normal levels since treatment. There are no new clinical signs and symptoms.

## Discussion

Kaposiform hemangioendothelioma is a rare vascular neoplasm that mostly appears in infancy or early childhood. KHE is a kind of intermediate tumor type with locally aggressive characteristics, which is characterized by dysregulation of angiogenesis and lymphangiogenesis. KHE may develop into life-threatening thrombocytopenia and consumptive coagulopathy, known as KMP. Approximately 12% of KHE patients manifested as deep masses (3). Intrathoracic and retroperitoneal lesions are more likely to develop KMP because they are more expansive and infiltrative.

Deep KHEs, especially visceral KHEs, are usually undetectable, easily missed clinically, and generally associated with high mortality. The co-existence of a vascular mass with prominent thrombocytopenia and coagulopathy, including hypofibrinogenemia, can make the diagnosis of KHE relatively straightforward. In patients with deep KHE without KMP, a definitive diagnosis often depends on biopsy because of the non-specific and vague symptoms. A definitive preoperative differential diagnosis between deep KHE and a malignant tumor (e.g., neuroblastoma or sarcoma) is also complex and challenging in patients with spine involvement. Imaging findings are significant for pre-treatment diagnosis, but a combination of clinical features, laboratory, and pathological assessments are essential for diagnosis. KHE should be part of the differential diagnosis in an infant presenting with purpura and unexplained profound thrombocytopenia and coagulopathy.

In this case, ^18^F-FDG PET/CT has played an important part. PET/CT showed a low metabolic activity within the retroperitoneal mass, which greatly helped physicians rule out a malignant tumor. After comprehensive consideration of the patient’s PET/CT imaging and clinical findings, the patient was given sirolimus monotherapy rather than combination therapy. The good treatment response of the patient proved this judgment was feasible. There are few reports about ^18^F-FDG PET/CT in KHE. Xu et al. (4) reported a 37-year-old woman with KHE in the sacrum, who showed intense ^18^F-FDG accumulation. Another KHE associated with the lymphangiomatosis case involving mesentery and ileum demonstrated mild ^18^F-FDG uptake in the lesion (5). This may indicate that many factors, such as age at disease onset, location, and disease activity can affect the functional pattern of KHE in PET/CT. In addition, the biopsy is frequently not possible or recommended in KHE with severe KMP, so accurate non-invasive examination becomes more important. PET/CT, which can reflect both morphological and functional characteristics, may be an ideal choice.

In this case, although the lesion was located in the retroperitoneum, the patient did not display any symptoms of KMP on admission: her platelet count was normal, and no obvious skin lesions were observed. The patient was diagnosed with immune thrombocytopenia at 7-month age with a minimum platelet count of 38 × 10^9^/L, which is consistent with previous research on KMP (3). According to her parent, her face and legs used to appear petechial hemorrhages. The patient’s platelet counts gradually return to a normal level without any special treatment, and her skin lesions disappeared. We presume that this patient has a long disease duration (probably from birth) and her KHE lesion’s activity decreased compared with the most active period. Imaging findings confirmed what we thought. ^18^F-FDG PET/CT showed very mild uptake elevation of the retroperitoneal lesion, suggestive of low disease activity. In addition, sclerotic bone lesions and long-term symptoms suggested a long disease course. The basic pathophysiology of KMP is platelet trapping, activation, and consumption within the abnormal vascular structure. With reduced disease activity, the platelet count may return to a normal level but KHE lesions seldom spontaneously regress (6). It was reported that the signs and symptoms of congenital KHE with KMP may alleviate spontaneously, but the lesions would rebound accompanying severe KMP within the next few days or weeks (1). The incidence of KMP in KHE with deep lesions is probably underestimated because the platelet count may have returned to normal by the time the lesion is discovered.

The aggressive characteristics and destructive growth patterns of KHE can cause functional limitations and pain in joints and muscles, which may affect patients’ abilities to perform routine daily activities (7). KMP usually causes acute pain at the tumor sites, and musculoskeletal disorders are frequently found in cases involving the extremities. Lesions in thoracic or retroperitoneal may cause scoliosis (8). Early diagnosis and aggressive treatment are important for these patients. Musculoskeletal disorders may cause by tumor infiltration of the muscles, connective tissues, and joint structures, which can lead to diffuse muscle fibrosis and joint contracture (9). Another cause of functional limitations or pain is local nerve compression. Some studies suggested in most patients, the muscle lesions were still present even after long-term treatment, which is often inconsistent with stabilization of lesions and/or hematological parameters (7, 10). In this case, ^18^F-FDG PET/CT provided information about the glucose metabolism and morphology of muscles. Around the tumor, enlarged muscles and bone destruction were found with slight FDG uptake. The width of psoas on the affected side was 3.2 cm (SUVmax 1.4) whereas that on the healthy side was only 1.8 cm (SUVmax 0.9), suggesting the possibility of evasion of psoas and spine around the tumor. After 6-month sirolimus treatment, the patient’s symptoms in her left leg had a remarkable improvement and almost disappeared. Thus, nerve compression may be the main cause of her symptom. Low activity of lesions and a rapidly shrinking mass correlated with her good prognosis.

The treatment options for KHE are not standardized due to the marked heterogeneity and rarity of KHE. Considering the low platelet level in patients with KHE and the unclear boundary of the lesion, surgical treatment is difficult. In 2013, a consensus treatment statement for KHE was published (11), but the recommendation is based on expert opinion rather than rigorous clinical studies. Pharmacological treatments have improved in recent years, possible options include vincristine, corticosteroids, sirolimus, interferon-α, etc. (1). Notably, an individualized treatment plan should be given to different patients. Monotherapy is usually not recommended in patients with KMP. In recent years, an increasing number of studies have reported the application of mTOR inhibitors sirolimus in KHE (12–14). It has shown good therapeutic results, and sirolimus therapy also exhibited a high response rate (94%) in patients who did not respond to corticosteroids or vincristine (15). According to previous reports, short-term prednisolone treatment plus sirolimus therapy was superior to sirolimus monotherapy in improving the signs and symptoms of active KHE with KMP (16). But for patients with milder symptoms or less activity, monotherapy may be sufficient. It may be the development direction of KHE treatment that comprehensive consideration of PET characteristics and clinical manifestations to develop a personalized treatment plan.

## Conclusion

In conclusion, this case shows a rare deep KHE without an abnormal platelet count and with a good response to sirolimus monotherapy. This case demonstrated the advantage of ^18^F-FDG PET/CT in the evaluation of disease activity in KHE and the possibility of using ^18^F-FDG PET/CT to guide therapy and prognostication.

## Data availability statement

The original contributions presented in this study are included in the article/supplementary material. Further inquiries can be directed to the corresponding author.

## Ethics statement

The study was approved by the Institutional Review Board of Peking University First Hospital. Written informed consent was obtained from the minor’s legal guardian for the publication of any potentially identifiable images or data included in this article.

## Author contributions

YQ: acquisition and analysis of the work, draft the manuscript, imaging data collection, and analysis. WH and ZC: manuscript editing. QY and LS: formal analysis and resources. LK and YF: supervision and writing—review and editing. All authors met the requirements for authorship for the submitted version and agreed to its submission.

## References

[1] Ji Y Chen S Yang K Xia C Li L. Kaposiform hemangioendothelioma: current knowledge and future perspectives. *Orphanet J Rare Dis.* (2020) 15:39. 10.1186/s13023-020-1320-1 32014025 PMC6998257

[2] Putra J Gupta A. Kaposiform haemangioendothelioma: a review with emphasis on histological differential diagnosis. *Pathology.* (2017) 49:356–62. 10.1016/j.pathol.2017.03.001 28438388

[3] Ji Y Yang K Peng S Chen S Xiang B Xu Z Kaposiform haemangioendothelioma: clinical features, complications and risk factors for kasabach-merritt phenomenon. *Br J Dermatol.* (2018) 179:457–63. 10.1111/bjd.16601 29603128 PMC11032113

[4] Xu H Song L Duan J. 18f-Fdg Pet/Ct findings in a woman with kaposiform hemangioendothelioma in the sacrum. *Clin Nucl Med.* (2022) 47:e353–4. 10.1097/rlu.0000000000004000 35020652

[5] Dong A Zhang L Wang Y He T Zuo C. Abdominal kaposiform hemangioendothelioma associated with lymphangiomatosis involving mesentery and ileum: a case report of Mri, Ct, and 18f-Fdg Pet/Ct Findings. *Medicine.* (2016) 95:e2806. 10.1097/md.0000000000002806 26871848 PMC4753944

[6] O’Rafferty C O’Regan GM Irvine AD Smith OP. Recent advances in the pathobiology and management of kasabach-merritt phenomenon. *Br J Haematol.* (2015) 171:38–51. 10.1111/bjh.13557 26123689

[7] Schaefer BA Wang D Merrow AC Dickie BH Adams DM. Long-term outcome for kaposiform hemangioendothelioma: a report of two cases. *Pediatr Blood Cancer.* (2017) 64:284–6. 10.1002/pbc.26224 27701822

[8] Qiu T Yang K Dai S Chen S Ji Y. Case report: kaposiform hemangioendothelioma with spinal involvement. *Front Pediatr.* (2021) 9:600115. 10.3389/fped.2021.600115 33912518 PMC8071878

[9] Ji Y Yang K Chen S Peng S Lu G Liu X. Musculoskeletal complication in kaposiform hemangioendothelioma without kasabach-merritt phenomenon: clinical characteristics and management. *Cancer Manag Res.* (2018) 10:3325–31. 10.2147/cmar.S171223 30233248 PMC6135070

[10] Enjolras O Mulliken JB Wassef M Frieden IJ Rieu PN Burrows PE Residual lesions after kasabach-merritt phenomenon in 41 patients. *J Am Acad Dermatol.* (2000) 42(2 Pt 1):225–35. 10.1016/s0190-9622(00)90130-0 10642677

[11] Drolet BA Trenor CC III Brandão LR Chiu YE Chun RH Dasgupta R Consensus-derived practice standards plan for complicated kaposiform hemangioendothelioma. *J Pediatr.* (2013) 163:285–91. 10.1016/j.jpeds.2013.03.080 23796341

[12] Boccara O Puzenat E Proust S Leblanc T Lasne D Hadj-Rabia S The effects of sirolimus on kasabach-merritt phenomenon coagulopathy. *Br J Dermatol.* (2018) 178:e114–6. 10.1111/bjd.15883 28796887

[13] Zhang G Chen H Gao Y Liu Y Wang J Liu XY. Sirolimus for treatment of kaposiform haemangioendothelioma with kasabach-merritt phenomenon: a retrospective cohort study. *Br J Dermatol.* (2018) 178:1213–4. 10.1111/bjd.16400 29388191

[14] Lackner H Karastaneva A Schwinger W Benesch M Sovinz P Seidel M Sirolimus for the treatment of children with various complicated vascular anomalies. *Eur J Pediatr.* (2015) 174:1579–84. 10.1007/s00431-015-2572-y 26040705

[15] Peng S Yang K Xu Z Chen S Ji Y. Vincristine and sirolimus in the treatment of kaposiform haemangioendothelioma. *J Paediatr Child Health.* (2019) 55:1119–24. 10.1111/jpc.14370 30604513

[16] Ji Y Chen S Zhou J Yang K Zhang X Xiang B Sirolimus plus prednisolone vs sirolimus monotherapy for kaposiform hemangioendothelioma: a randomized clinical trial. *Blood.* (2022) 139:1619–30. 10.1182/blood.2021014027 35030255

